# Coronavirus RNA-dependent RNA polymerase interacts with the p50 regulatory subunit of host DNA polymerase delta and plays a synergistic role with RNA helicase in the induction of DNA damage response and cell cycle arrest in the S phase

**DOI:** 10.1080/22221751.2023.2176008

**Published:** 2023-02-15

**Authors:** Li Quan, Xinxin Sun, Linghui Xu, Rui Ai Chen, Ding Xiang Liu

**Affiliations:** a Zhaoqing Branch Center of Guangdong Laboratory for Lingnan Modern Agricultural Science and Technology, Zhaoqing, China; b Integrative Microbiology Research Centre, South China Agricultural University, Guangzhou, China

**Keywords:** Coronavirus, nsp12, DNA polymerase delta p50 subunit, interaction, cell cycle arrest

## Abstract

Disruption of the cell cycle is a common strategy shared by many viruses to create a conducible cellular microenvironment for their efficient replication. We have previously shown that infection of cells with gammacoronavirus infectious bronchitis virus (IBV) activated the theataxia-telangiectasia mutated (ATM) Rad3-related (ATR)/checkpoint kinase 1 (Chk1) pathway and induced cell cycle arrest in S and G2/M phases, partially through the interaction of nonstructural protein 13 (nsp13) with the p125 catalytic subunit of DNA polymerase delta (pol δ). In this study, we show, by GST pulldown, co-immunoprecipitation and immunofluorescent staining, that IBV nsp12 directly interacts with the p50 regulatory subunit of pol δ in vitro and in cells overexpressing the two proteins as well as in cells infected with a recombinant IBV harbouring an HA-tagged nsp12. Furthermore, nsp12 from severe acute respiratory syndrome coronavirus (SARS-CoV) and SARS-CoV-2 was also able to interact with p50. These interactions play a synergistic role with nsp13 in the induction of S phase arrest. The fact that subunits of an essential cellular DNA replication machinery physically associate with two core replication enzymes from three different coronaviruses highlights the importance of these associations in coronavirus replication and virus-host interaction, and reveals the potential of targeting these subunits for antiviral intervention.

## Introduction

Coronaviruses are major human and animal pathogens, causing serious public health concerns and huge economic losses. Fatal coronavirus infections, such as severe acute respiratory syndrome coronavirus (SARS-CoV), Middle East respiratory coronavirus (MERS-CoV) and SARS-CoV-2, have an enormous negative impact on social and economic development [1,2]. Avian gammacoronavirus infectious bronchitis virus (IBV) is a major avian pathogen, causing an acute, highly contagious infection affecting all ages and types of chickens [3]. Coronaviruses are a group of enveloped viruses with single-stranded and positive-sense RNA genome. The genome encodes polyprotein 1a (pp1a) and 1ab (pp1ab) [1], which are cleaved by virus-encoded proteinases into 16 or 15 nonstructural proteins (nsps). Among them, nsp12 and nsp13 are viral RNA-dependent RNA polymerase (RdRP) and RNA helicase, essential for viral RNA replication and transcription [4,5].

The cell cycle or cell division cycle is a highly regulated process that maintains normal metabolism and prevents damaged DNA from being inherited, and can be controlled by intracellular and extracellular factors [6]. The eukaryotic cell cycle is generally divided into four phases: gap 1 (G1), synthesis (S), gap 2 (G2) and mitosis (M). Coronavirus employs different strategies to deregulate cell cycle checkpoints and modulate cellular proliferation pathways to obtain cellular factors required for efficient replication [7]. For example, mouse hepatitis virus (MHV) induces cell cycle arrest in G0/G1 phase, thereby obtaining the ribonucleotides required for the synthesis of large amounts of RNA, while facilitating cell replication and preventing cell death [8]. Transmissible gastroenteritis virus (TGEV) infection induces S and G2/M arrest, aiming to establish a pseudo-S-phase state for the virus to replicate its genome [9]. IBV infection also induces S and G2/M arrest [10,11], partly through the interaction of nsp13 with the p125 catalytic subunit of mammalian DNA polymerase delta (pol δ) [12].

Pol δ is a four-subunit, p125/p50/p68/p12, enzyme that plays crucial multifunctional roles in DNA replication and various DNA repair processes [13]. As replication stress or genotoxic agents trigger degradation of the p12 subunit, pol δ is converted from a heterotetramer (p125/p50/p68/p12) to a trimer lacking the p12 subunit (p125/p50/p68) [14]. This transformed trimer changes the properties of the enzyme, becoming less active for trans-lesion synthesis in the presence of DNA base damage, while having greater proofreading ability for insertion of erroneous nucleotides and extension of mismatched primers, with enhanced ability to detect primers and primer errors [15].

Previous studies have shown that IBV and SARS-CoV nsp13 interacts with p125 to significantly induce S-phase arrest, suggesting that nsp13 may inhibit DNA replication at replication forks [12]. In this study, nsp12 from IBV, SARS-CoV and SARS-CoV-2 was demonstrated to interact with the p50 regulatory subunit of pol δ. This interaction plays a synergistic role with nsp13 in the induction of cell cycle arrest in the S phase.

## Experimental procedures

### Cell culture and viral infection

293  T, HeLa, H1299 and DF-1 cells were cultured in DMEM medium (Gibco) supplemented with 10% FBS and 1% penicillin-streptomycin (Gibco), at 37°C supplied with 5% CO_2_. The egg-adapted Beaudette strain of IBV (ATCC VR-22) was obtained from the American Type Culture Collection (ATCC) and adapted to Vero cells (IBV-p65) as previously described [16,17]. To prepare viral stocks, monolayers of Vero cells were seeded with IBV-p65 at a multiplicity of infection (MOI) of approximately 0.1 and incubated at 37°C for 24 h. After three freeze/thaw cycles, cell lysates were clarified by centrifugation at 1500 g for 30 min at 4°C, supernatants were aliquoted and stored at −80°C as viral stocks. The titer of virus stock was determined by plaque assay. The recombinant IBV carrying an HA-tagged nsp12 (rIBV-HA-RdRP) was rescued, as previously described [18], using a reverse genetics system based on IBV-p65.

For IBV infection experiments, cells seeded on 6-well plates were washed twice with PBS unless otherwise stated, infected with IBV at a multiplicity of infection (MOI) of approximately 2 or incubated with an equal volume of mock lysates. After 1 h of adsorption, the medium was discarded, and the cells were washed twice and incubated in serum-free medium at 37°C until harvest.

### Construction of plasmids

Plasmid XJ40-Flag or pXJ40-Myc were used for cloning coronavirus nsp12 from IBV, SARS-CoV and SARS-CoV-2. IBV nsp12 was obtained using the RT kit (Takala) with the specific primer: TGATTCACTTAGACAACCAAAATCTTCTGTTCAA for the first strand synthesis, and primer pairs GACGATGATAAGTCCGGATCCTTTAAACGGGTACGGGG and CCCCGTACCCGTTTAAAGGATCCGGACTTATCATCGTC for PCR amplification. The fragment was cloned into pXJ-Flag. SARS-CoV nsp12 (NC_004718.3) and SARS-CoV-2 nsp12 (NC_045512.2) were synthesized by GeneWise gene synthesis service and inserted into pXJ40-Flag.

The cDNA for p50 was amplified by PCR with the genomic DNA extracted from H1299 cells using primer pairs GTTGCGGGAAACATATGGGATCCATGTTTTCTGAGCAGGCTGCCCAG and CCGAGCTCCTGCAGCTCGAGTCAGGGGCCCAGCCCCAG. The PCR fragment was cloned into pXJ-Myc. Plasmid GEX-p50C was constructed by cloning the PCR fragment encoding the C-terminal 172 amino acids of p50 (235–469 amino acids) into pGEX-5X-3.

### GST-pull down

Plasmid GEX-5X1 and pGEX-p50C, respectively, were transformed into *E. coli* BL21(DE) and overexpression of GST and GST-p50c fusion proteins was accomplished by induction with 400 μM isopropyl 1-thio-β-d-galactopyranoside (IPTG) for 3 h at 37°C. The proteins were purified using a GST purification module (Amersham Biosciences) according to the manufacturer's instructions.

Expression of ^35^S-labelled nsp12 in wheat germ extract by in vitro translation in the presence of [^35^S] methionine was carried out according to the recommended protocol (Promega). In the pulldown experiment, 30 μL of GST-Sepharose 4B or GST-p50C-Sepharose 4B Microbeads were added with 90 μL of lysis buffer (140 mM NaCl, 10 mM Tris-HCl (pH 8.0) and 0.5% Nonidet P-40)) for 1 h at room temperature. Sepharose 4B beads were washed 5 times with the lysis buffer and boiled in 2x SDS loading buffer for 7 min. The eluted pellets were then subjected to SDS-PAGE.

### Co-immunoprecipitation and Western blotting

Cells were transfected with plasmid DNA using ExFect Transfection Reagent (Vazyem). At 24 h post-transfection, cells were lysed with ice-cold RIPA Lysis Buffer (50 mM Tris [pH 7.4], 150 mM NaCl, 1% NP-40, 0.5% sodium deoxycholate, 0.1% SDS) with 1 µL/mL protease inhibitor cocktail (Beyotime) at 4°C for 20 min. After clarified by centrifugation at 12,000 rpm for 10 min, cell lysates were immunoprecipitated with EZview Red anti-HA agarose affinity gel (Sigma-Aldrich), anti-Flag or anti-Myc affinity gel (Beyotime) as indicated for 4 h at 4°C, and washed five times with lysis buffer. Total cell lysates and precipitates were boiled with sample buffer, separated by sodium dodecyl sulphate-polyacrylamide gel electrophoresis (SDS-PAGE), and transferred to nitrocellulose membranes. The membranes were blocked with 5% nonfat milk for 1 h at room temperature and blotted with specific antibodies.

### Flow cytometry

Infected or transfected cells were harvested at predetermined times and fixed with 70% cold ethanol at −20°C overnight. After washing once with phosphate-buffered saline (PBS), 500 µL of FxCycle™ PI/RNase Staining Solution (Thermo Fisher Scientific) was added and incubated at room temperature for 30 min in the dark. Cell cycle distribution was measured by propidium iodide (PI) staining using a CytoFLEX flow cytometer (Beckman Coulter). Data were analysed using ModFit LT 5.0 (Verity Software House).

### Immunofluorescence and confocal microscopy

H1299 cells grown on glass bottom dishes were infected for indicated times, fixed with 4% paraformaldehyde for 20 min, and treated with 0.25% Triton X-100 in PBS for 10 min. After washing 3 times with PBS for 10 min and blocking with 5% bovine serum albumin in PBS for 2 h, cells were incubated with the indicated antibodies overnight at 4°C, and the corresponding fluorescent secondary antibodies were co-incubated at room temperature for 30 min. After washing, cells were stained with DAPI (Sigma), and images were acquired with a fluorescence microscope (or a confocal microscope).

### Statistical analysis

The one-way ANOVA method was used to analyse the significant difference between the indicated sample and the respective control sample. Significance levels were presented by the *p*-value (ns, non-significant; *, *p* < 0.05; **, *p* < 0.01; ***, *p* <0.001; ****, *p* < 0.0001).

## Result

### IBV nsp12 directly interacts with the p50 subunit of DNA Pol δ

**Figure 1.**
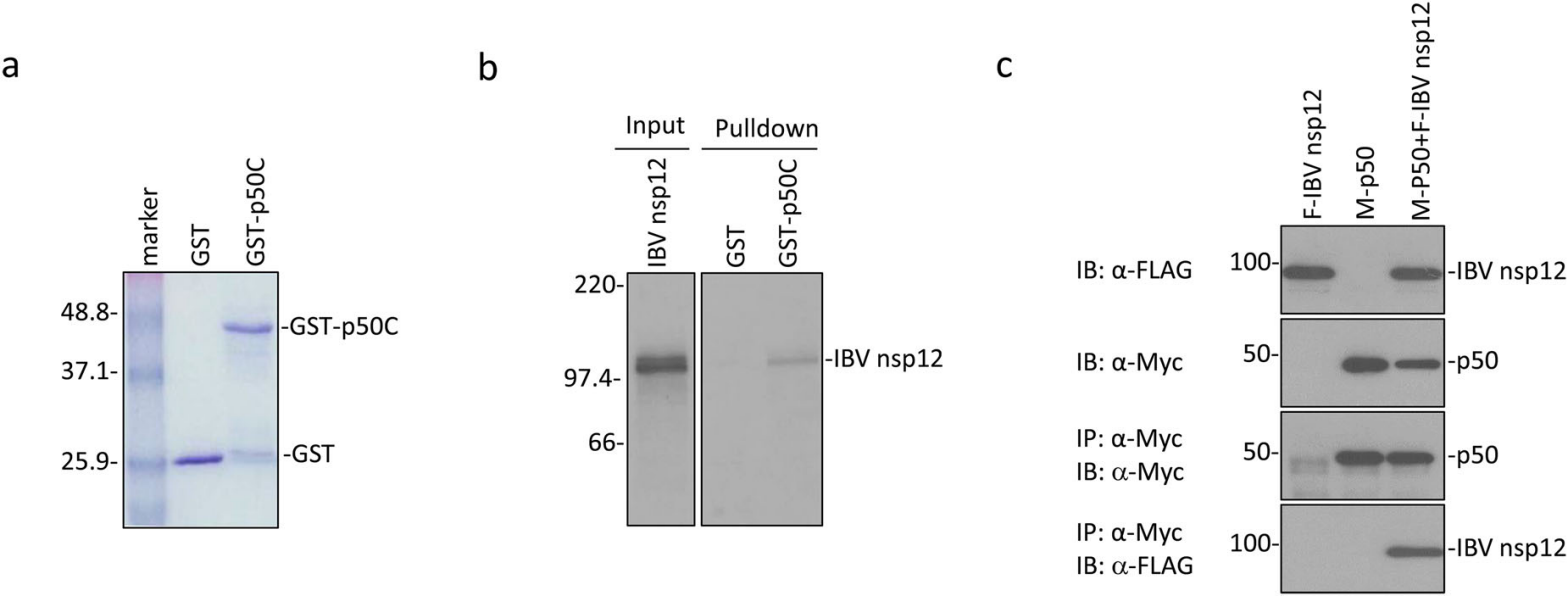
Interaction of IBV nsp12 with p50 in vitro and in cells overexpressing the two proteins. (a) Expression and purification of GST-p50C fusion protein. GST and GST-p50C fusion protein were expressed in *E. coli* and partially purified with glutathione-Sepharose 4B beads. The proteins were separated on an SDS-12% polyacrylamide gel and visualized by Coomassie brilliant blue staining. Numbers on the left indicate sizes in kilodalton. (b) Interaction of IBV nsp12 with p50C by GST pulldown assay. GST and GST-p50C were used to pull down the ^35^S-labeled in vitro translated nsp12. Precipitates and in vitro translated products were detected by autoradiography, and GST protein was used as a negative control. Numbers on the left indicate sizes in kilodalton. (c) Interaction of IBV nsp12 with p50 in cells overexpressing the two proteins. 293  T cells were transfected with Flag-tagged IBV nsp12 (F-IBV nsp12) and Myc-tagged p50 (M-p50) either alone or together. Cells were harvested at 24 h post-transfection, lysed with RIPA buffer, and subjected to immunoprecipitation with anti-Myc-coated beads. Total cell lysates (top two panels) and precipitates (bottom two panels) were immunoblotted using anti-Flag and anti-Myc antibodies, respectively. Numbers on the left indicate sizes in kilodalton.

The p50 subunit of DNA pol δ as a potential partner of IBV nsp12 was initially identified by yeast two-hybrid screening [19]. The C-terminal 172 amino acid region of p50 (p50C) was shown to interact with a bait containing the IBV genomic sequence from nucleotides 14,129–15,002 coding for 291 amino acids (amino acids 607–897) in the C-terminal region of nsp12. This potential interaction was first investigated by GST pulldown assay. GST and GST-p50C fusion protein were expressed in bacteria and pulled down with glutathione-Sepharose 4B beads (Figure 1(a)). Co-precipitation of GST-p50C and GST with the in vitro synthesized IBV nsp12 showed that only GST-p50 could be co-precipitated with IBV nsp12, while GST alone could not interact with the protein (Figure 1(b)).

The interaction between IBV nsp12 and p50 was then tested by co-immunoprecipitation of the two proteins overexpressed in mammalian cells. To facilitate the detection of their expression, the N-termini of p50 and IBV nsp12 were tagged with a Myc and a Flag tag, respectively. The two proteins were efficiently expressed either alone or together in 293  T cells (Figure 1(c)). Immunoprecipitation with anti-Myc beads showed that IBV nsp12 could be co-precipitated only when the two proteins were co-expressed (Figure 1(c)). These results confirm the direct interaction of IBV nsp12 with p50 in the absence of other viral proteins and viral RNA.

Immunofluorescent staining was performed in HeLa cells overexpressing the Flag-tagged IBV nsp12 with monoclonal anti-Flag antibody at 24 h post-transfection. A majority of the Flag-tagged nsp12 protein was found to be localized to the perinuclear region (Supplementary Figure 1a). However, a certain proportion of the protein was also localized in the nucleus and other cytoplasmic regions (Supplementary Figure 1b). Co-expression of the Flag-tagged IBV nsp12 and the Myc-tagged p50 showed significant overlapping of the two images in the perinuclear, nuclear and cytoplasmic regions, further supporting the direct interaction of the two proteins in the transfected cells.

### Interaction of IBV nsp12 with p50 induces DNA damage response and cell cycle arrest in the S phase

**Figure 2.**
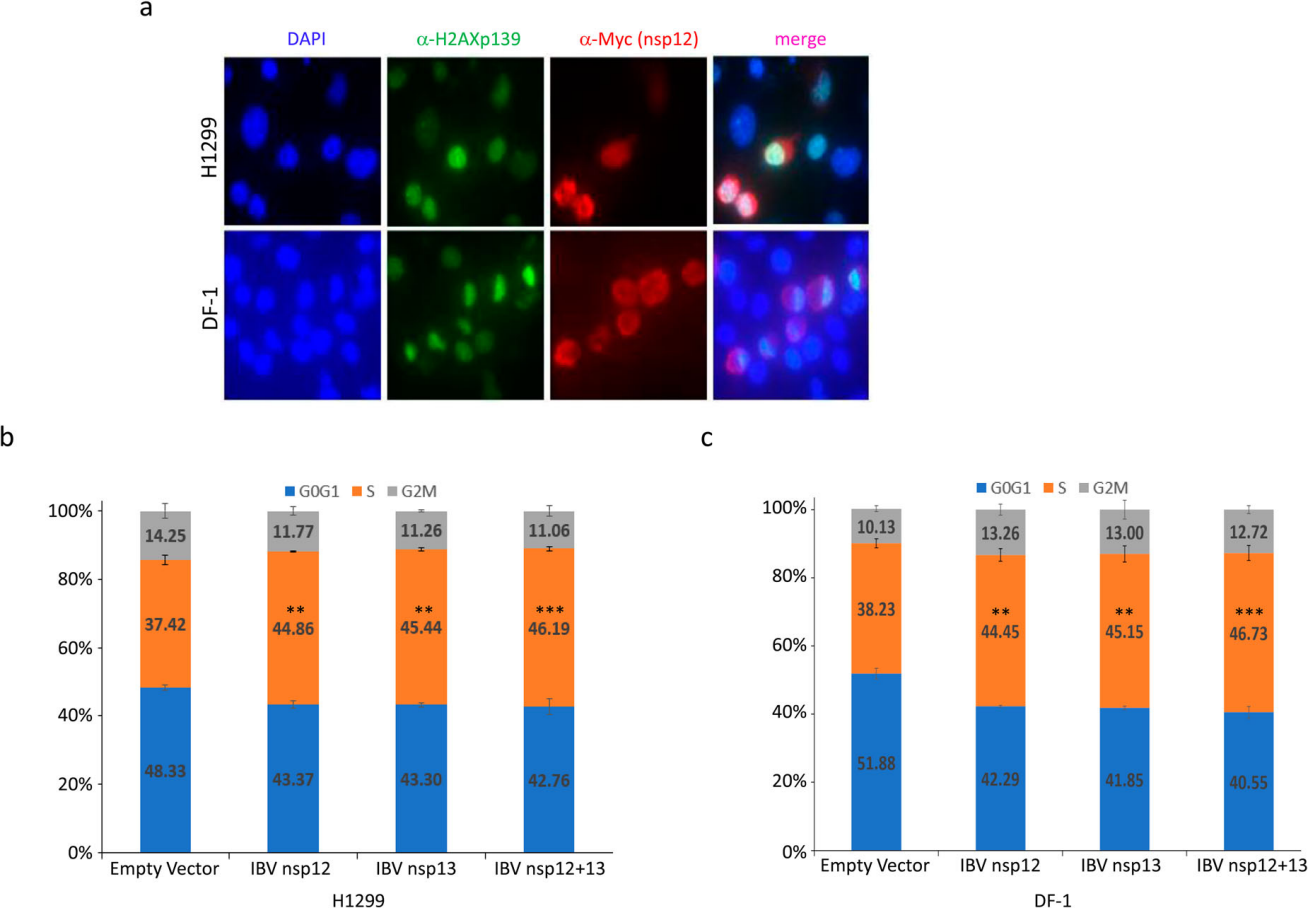
Induction of DNA damage response and cell cycle arrest in S phase in cells overexpressing IBV nsp12. (a) Induction of DNA damage response in cells overexpressing IBV nsp12. H1299 and DF-1 cells were transfected with Myc-tagged IBV nsp12, fixed at 48 h post-transfection, stained with DAPI and co-immunostained with mouse anti-Myc and rabbit anti-γH2AX antibodies, and examined by microscopy. (b) Induction of S-phase arrest in cells overexpressing IBV nsp12. Hl299 cells transfected with IBV nsp12 and nsp13 either alone or together were fixed at 24 h post-transfection and stained with PI. Cell cycle profiles were determined by flow cytometry. Data were analysed by using ModFit LT 5.0 software to determine the percentage of cells at each stage of the cell cycle in asynchronously growing cells. Results are presented as three replicate experiments. Significance levels were presented by the *p*-value (**, *p* < 0.01; ***, *p* < 0.001). (c) Induction of cell cycle arrest in DF-1 cells overexpressing IBV nsp12. DF-1 cells transfected with IBV nsp12 and nsp13 either alone or together were fixed at 24 h post-transfection and analysed as in (b). Results are presented as three replicate experiments. Significance levels were presented by the *p*-value (**, *p* < 0.01; ***, *p* < 0.001).

In higher eukaryotic cells, double-strand breaks in chromosomal DNA rapidly initiate phosphorylation of histone H2A variant H2AX at serine 139 to produce γ-H2AX [20,21]. Our previous study demonstrated that IBV infection of Vero and H1299 cells induced γH2AX, which can be used as a reliable marker for the induction of DNA replication stress response and cell cycle arrest in cells transfected with coronavirus nsp12 [12]. In this study, γH2AX was used as a marker to demonstrate if the interaction between IBV nsp12 and p50 would induce the DNA damage response. Immunofluorescent staining of H1299 cells overexpressing a Myc-tagged IBV nsp12 demonstrated that, once again, the protein was mainly expressed in the cytoplasm, with certain enrichment in the perinuclear region (Figure 2(a)). Staining the same cells with anti-γH2AX antibody revealed much brighter nuclear staining of cells overexpressing IBV nsp12 (Figure 2(a)). Overlay of the two images showed the localization of a certain proportion of the Myc-tagged IBV nsp12 in the nuclei of the transfected cells (Figure 2(a)). Very similar fluorescent images were also obtained in DF-1 cells transfected with the Myc-tagged IBV nsp12 (Figure 2(a)), confirming the induction of DNA damage response in both human cancer cells and chicken embryo fibroblast cells.

The effect of IBV nsp12 overexpression on cell cycle progression was then tested in H1299 and DF-1 cells expressing nsp12 and nsp13 either alone or together, by flow cytometry after staining the nuclear DNA with PI. When IBV nsp12 or nsp13 was expressed alone, the S-phase contents of cells were increased by approximately 7–9%, compared to those in control cells transfected with the empty plasmid (Figure 2(b,c)). When the two proteins were co-expressed, moderately more cells were arrested in the S phase, compared to cells transfected with either protein alone (Figure 2(b,c)). Statistical analysis demonstrated that these differences are significant (Figure 2(b,c)). These data confirm that overexpression of IBV nsp12 induces cell cycle arrest in the S phase, which may be further induced by the co-expression of both nsp12 and nsp13 together.

### IBV nsp12 was mainly localized in the viral replication site in IBV-infected cells

**Figure 3.**
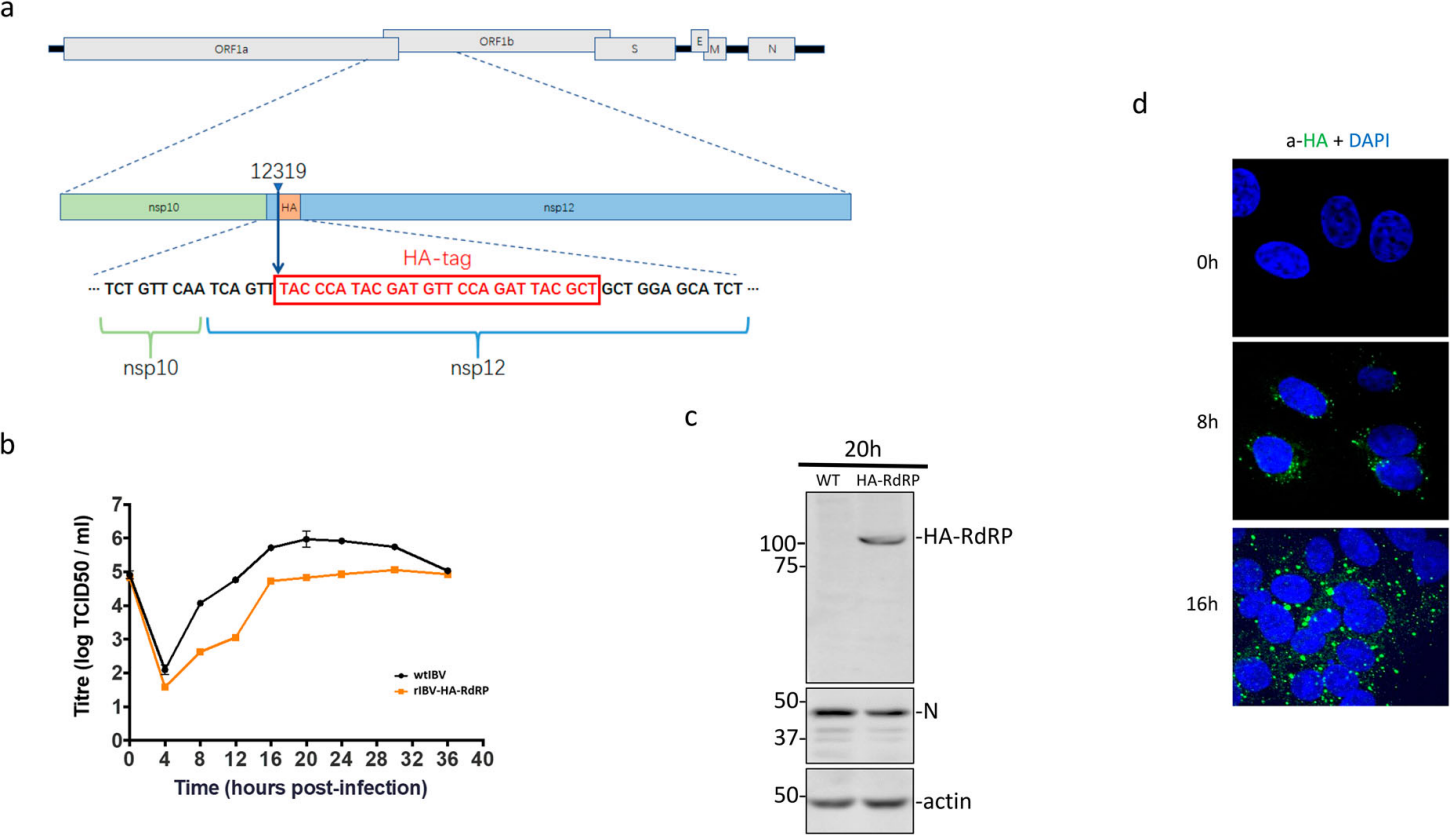
Subcellular localization of IBV nsp12. (a) Schematic diagram showing the genome structure of IBV and the position of the HA tag inserted in the recombinant IBV (rIBV-HA-RdRP) harbouring an HA-tagged RdRP. (b) Growth kinetics of rIBV-HA-RdRP. The growth kinetics of wild type (WT-rIBV) and rIBV-HA-RdRP were determined in cell lysates harvested at the indicated time points post-infection, using the TCID50 method. IBV titers are expressed in log TCID50 per millilitre. (c) Analysis of the HA-tagged RdRP (HA-nsp12) expression in H1299 cells infected with rIBV-HA-RdRP. Cells were infected with wild type IBV and rIBV-HA-RdRP at an MOI∼2, respectively, harvested at 20 h post-infection, and lysates prepared. Protein samples were separated by SDS-PAGE and analysed by Western blotted with anti-HA and anti-IBV N antibodies. β-Actin was used as a loading control. The sizes of the protein ladder in kDa are shown on the left. (d) Subcellular localization of the HA-tagged IBV nsp12 in infected cells. Vero cells were infected with rIBV-HA-RdRP at an MOI∼1, fixed at 0, 8 and 16 h post-infection, respectively, immunostained with mouse anti-HA antibody, and examined by confocal microscopy. Blue represents nuclei staining with DAPI and green represents HA-tagged nsp12.

The functional relevance of above observations was studied in IBV-infected cells. To precisely define the subcellular localization of nsp12 and its interaction with p50, a stable recombinant IBV, rIBV-HA-RdRP, containing an HA tag inserted at the nucleotide position 12319 in rIBV-p65, was constructed and successfully rescued (Figure 3(a)). Compared with wild type IBV (WT-rIBV), rIBV-HA-RdRP replicated more slowly in cell culture, with the peak titer approximately 10-fold lower than that of wild type virus (Figure 3(b)). Western blot analysis of infected cells using anti-N antibodies detected IBV N protein in cells infected with either wild type or rIBV-HA-RdRP, confirming the efficient replication of both viruses (Figure 3(c)). However, the HA-nsp12 (HA-RdRP) was detected only in cells infected with rIBV-HA-RdRP, but not in cells infected with WT-rIBV, confirming the efficient and stable expression of the HA-tagged nsp12 (Figure 3(c)).

The rescued rIBV-HA-RdRP was then used to study the subcellular localization of IBV nsp12 in IBV-infected cells by immunofluorescent staining with the monoclonal anti-HA antibody at 0, 8 and 16 h post-infection, respectively. Consistent with previous observations, a majority of the punctate HA-tagged nsp12 protein was found in the perinuclear region, the viral RNA synthesis site, in the infected cells (Figure 3(d)). However, a minor portion of the protein was also observed in the nucleus (Figure 3(d)).

The subcellular localization patterns in cells infected with rIBV-HA-RdRP were further explored by co-staining of the HA-tagged nsp12 and dsRNA at 0, 8, 16 and 36 h post-infection, respectively. The fluorescent images of dsRNA overlapped well with HA-nsp12 in the perinuclear region at 16 and 36 h post-infection (Supplementary Figure 2), confirming that a majority of the HA-tagged nsp12 is localized to the viral RNA replication site in IBV-infected cells and plays the main function as an RNA polymerase in viral replication. Meanwhile, the localization of a minor portion of the protein in the nucleus would support its function in regulating cell cycle progression.

### IBV nsp12 interacts with p50 in IBV-infected cells and induces aberrant cell cycle progression

**Figure 4.**
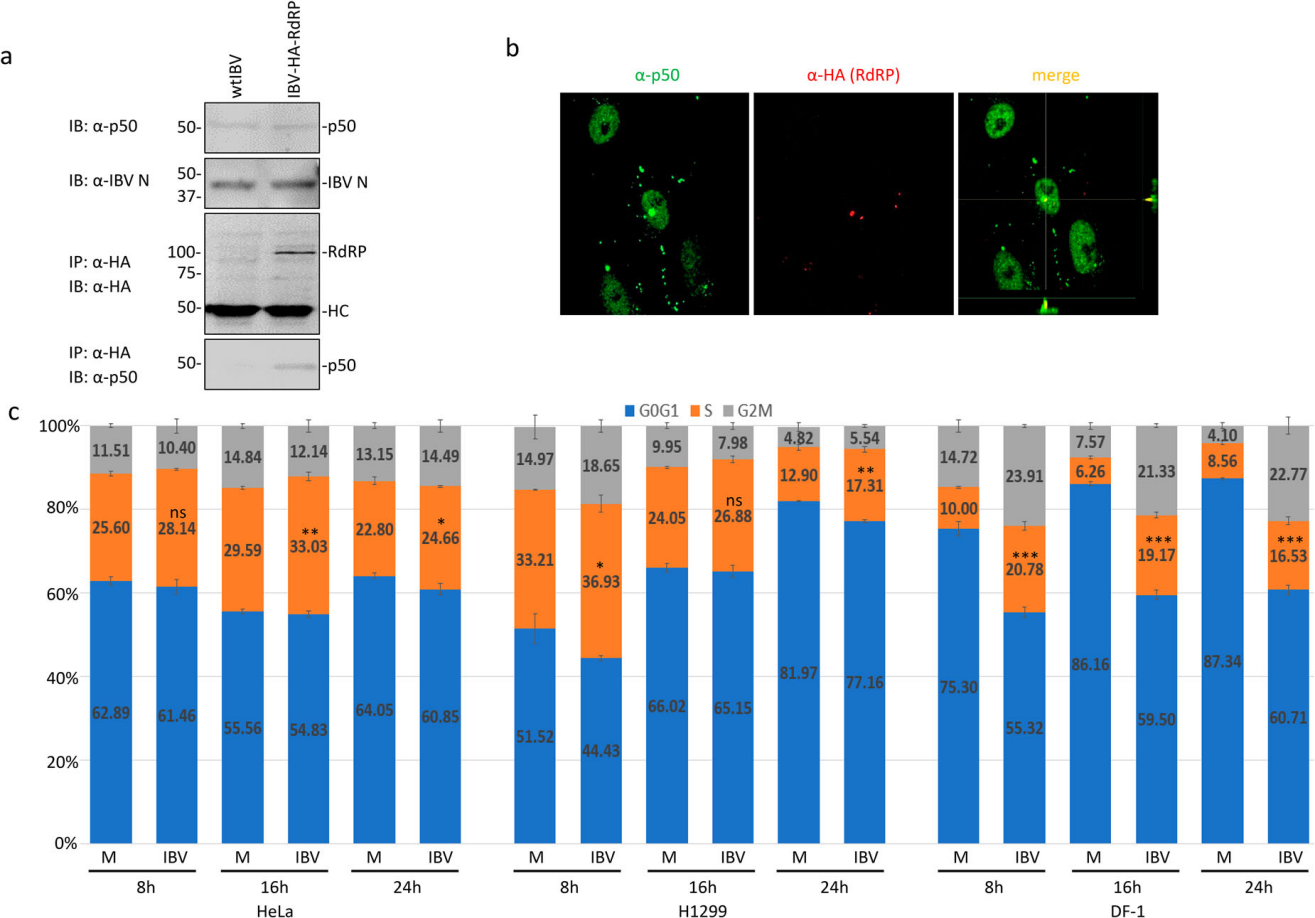
Interaction of IBV nsp12 with p50 in IBV-infected cells. (a) Co-precipitation of the endogenous p50 with HA-tagged nsp12 in cells infected with rIBV-HA-RdRP. H1299 cells were infected with wild type IBV (wtIBV) and rIBV-HA-RdRP at an MOI∼1, respectively, harvested at 10 h post-infection, and total cell lysates were prepared. Proteins in total cell lysates were either resolved directly on 8% SDS-PAGE or immunoprecipitated with anti-HA beads prior to loading on the gel. Immunoblotting was performed with anti-HA, anti-p50 and anti-IBV N antibodies, respectively. The sizes of the protein ladder in kDa are shown on the left. (b) Co-localization of p50 and HA-tagged nsp12 in the nuclei of IBV-infected cells. Vero cells were infected with rIBV-HA-RdRP, fixed at 10 h post-infection, and co-immunostained with mouse anti-p125 and rabbit anti-HA antibodies. (c) Induction of cell cycle arrest in IBV-infected DF-1, H1299 and HeLa cells. DF-1, H1299 and HeLa cells were infected with IBV at an MOI∼1, respectively, harvested at indicated times post-infection, and stained with PI for flow cytometry analysis. Data were analysed using ModFit LT 5.0 software to determine the percentage of cells at each stage of the cell cycle in asynchronously growing DF-1, H1299 and HeLa cells. Results are presented as three replicate experiments. Significance levels were presented by the *p*-value (ns, non-significance, *, *p* < 0.05; **, *p* < 0.01; ***, *p* < 0.001).

Interaction of IBV nsp12 with p50 in IBV-infected cells was studied in cells infected with wild type and rIBV-HA-RdRP, respectively. Western blot analysis detected similar amounts of IBV N protein and p50 in cells infected with either wild type or rIBV-HA-RdRP (Figure 4(a)). Immunoprecipitation with anti-HA antibody showed co-precipitation of the endogenous p50 subunit with the HA-tagged nsp12 in cells infected with rIBV-HA-RdRP, but not in cells infected with wild type IBV (Figure 4(a)).

The demonstration that IBV nsp12 did interact with the endogenous p50 in infected cells prompted further studies on the interaction between the two proteins by immunofluorescent staining. Double-staining of infected cells with antibodies against p50 and HA showed the overlapping images in the nucleus (Figure 4(b)). However, no obvious overlapping of the two images in the cytoplasm was observed, although a certain proportion of p50 was clearly detected in the cytoplasm (Figure 4(b)). These results indicate that the interaction between the two proteins may mainly occur in the nuclei of infected cells.

The effect of this interaction on cell cycle progression was further analysed in asynchronously growing H1299, HeLa and DF-1 cells infected with IBV by flow cytometry. In IBV-infected HeLa cells, significantly more S-phase cells (2–3%, 3–6% and 2% increases at 8, 16 and 24 h post-infection, respectively) were detected at most time points post-infection (Figure 4(c)). A similar cell cycle profile was also observed in IBV-infected H1299 cells (Figure 4(c)). It was also noted that slightly more accumulation of cells in the G2/M phase was detected in IBV-infected HeLa and H1299 cells at 24 h post-infection (Figure 4(c)). In IBV-infected DF-1 cells, a more drastic increase of S-phase and G2/M-phase cells was detected at all time points post-infection, compared with the mock-treated cells. These results demonstrate that the induction of S and G2/M phase arrest by IBV infection occurs in culture cells of three different origins.

### SARS-CoV and SARS-CoV-2 nsp12 interacts with p50 and induces cell cycle arrest in the S phase

**Figure 5.**
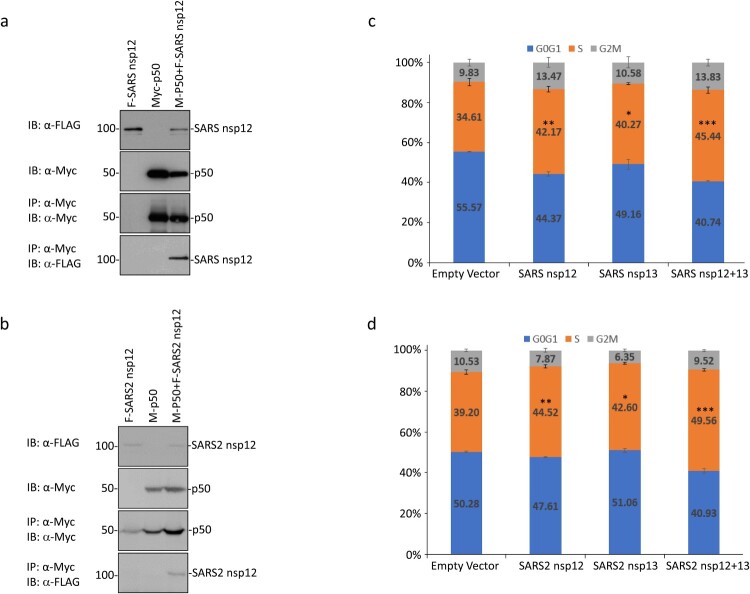
Interaction of SARS-CoV and SARS-CoV-2 nsp12 with p50 and induction of cell cycle arrest in S phase. (a) Interaction of SARS-CoV nsp12 with p50 in cells overexpressing the two proteins. 293  T cells were transfected with Flag-tagged SARS-CoV nsp12 (F-SARS nsp12) and Myc-tagged p50 (M-p50) either alone or together. Cells were harvested at 24 h post-transfection, lysed with RIPA buffer, and subjected to immunoprecipitation with anti-Myc-coated beads. Total cell lysates (top two panels) and precipitates (bottom two panels) were immunoblotted using anti-Flag and anti-Myc antibodies, respectively. Numbers on the left indicate sizes in kilodalton. (b) Interaction of SARS-CoV-2 nsp12 with p50 in cells overexpressing the two proteins. 293  T cells were transfected with Flag-tagged SARS-CoV-2 nsp12 (F-SARS2 nsp12) and Myc-tagged p50 (M-p50) either alone or together. Cells were harvested and analysed as in (a). (c) Induction of cell cycle arrest in cells overexpressing SARS-CoV nsp12. Hl299 cells transfected with SARS-CoV nsp12 and nsp13 either alone or together were fixed at 24 h post-transfection and stained with PI. Cell cycle profiles were determined by flow cytometry. Data were analysed using ModFit LT 5.0 software to determine the percentage of cells at each stage of the cell cycle in asynchronously growing cells. Results are presented as three replicate experiments. Significance levels were presented by the *p*-value (*, *p* < 0.05; **, *p* < 0.01; ***, *p* < 0.001) (d) Induction of cell cycle arrest in cells overexpressing SARS-CoV-2 nsp12. Hl299 cells transfected with SARS-CoV-2 nsp12 and nsp13 either alone or together were fixed at 24 h post-transfection and analysed as in (c). Results are presented as three replicate experiments. Significance levels were presented by the *p*-value (*, *p* < 0.05; **, *p* < 0.01; ***, *p* < 0.001).

Sequence comparison of the original IBV nsp12 region (from amino acids 607 to 897) used in the yeast two-hybrid screening with the corresponding sequences in SARS-CoV and SARS-CoV-2 nsp12 showed 64.20 and 64.90% amino acid identities, respectively (Supplementary Figure 3). In addition, long stretches of identical amino acids were also identified among the three proteins (Supplementary Figure 3). It suggests that SARS-CoV and SARS-CoV-2 nsp12 may also interact with p50. This possibility was first tested by transfection of 293  T cells with Flag-tagged SARS-CoV nsp12 and the Myc-tagged p50 either alone or together, and co-immunoprecipitation using anti-Myc beads. The results shown in Figure 5(a) confirmed the interaction between SARS-CoV nsp12 and p50. Similarly, SARS-CoV-2 nsp12 could also interact with p50 (Figure 5(b)).

Examination of the subcellular localization of the FLAG-tagged SARS-CoV and SARS-CoV-2 nsp12 overexpressed in HeLa cells by fluorescence microscopy demonstrated predominant perinuclear localization at 24 h post-transfection, similar to the patterns observed for IBV nsp12 (Supplementary Figure 1a). Co-expression of the Flag-tagged SARS-CoV and SARS-CoV-2 nsp12 with Myc-tagged p50, respectively, also showed a significant overlap of the two images in the perinuclear, nuclear and cytoplasmic regions (Supplementary Figure 1b).

Analysis of cell cycle profiles by flow cytometry demonstrated that overexpression of SARS-CoV and SARS-CoV-2 nsp12 also significantly induced cell cycle arrest in the S phase (Figure 5(c,d)). When co-expressed with their corresponding nsp13, significantly more cells arrested at the S phase were observed (Figure 5(c,d)), confirming that coronavirus nsp12 may play a synergistic role with nsp13 in the induction of cell cycle arrest in the S phase.

### A C-terminal conserved region in nsp12 is partially responsible for the interaction between nsp12 and p50

**Figure 6.**
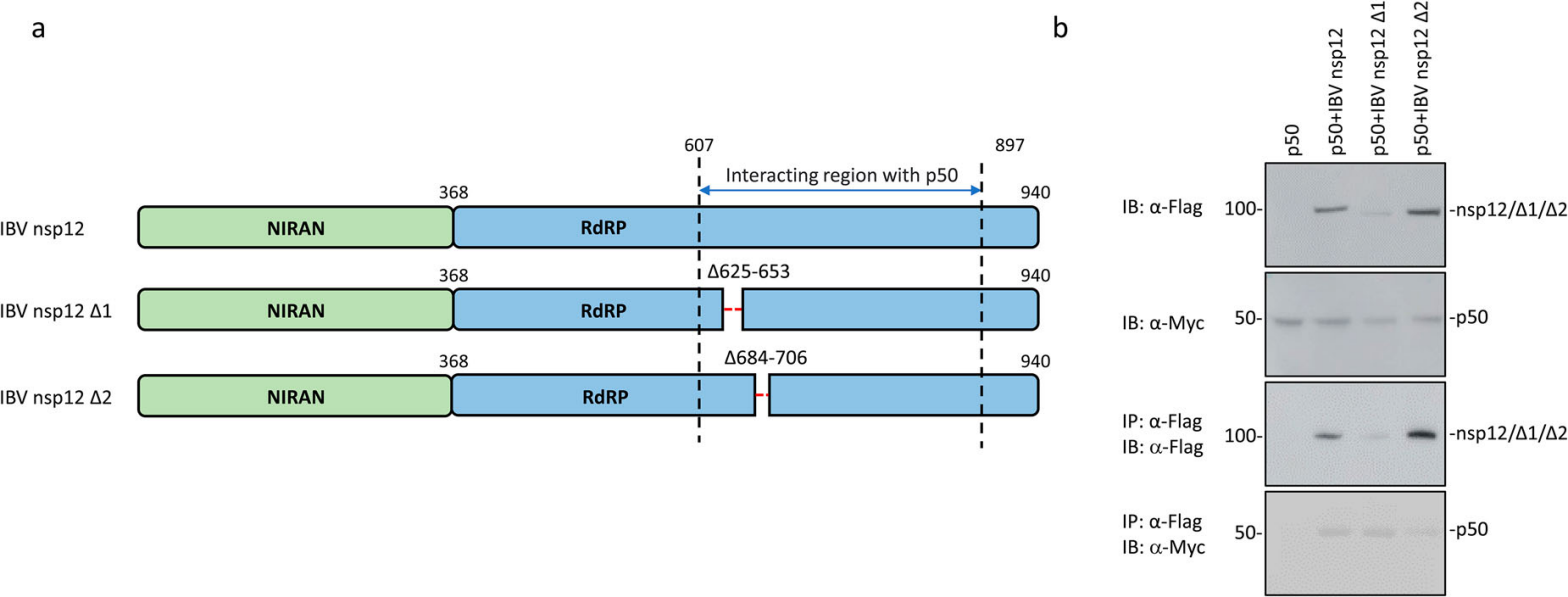
Identification of domain(s) in IBV nsp12 responsible for the interaction between nsp12 and p50. (a) Schematic diagram showing the construction of two deletion mutants of IBV nsp12, IBV nsp12 Δ1 and IBV nsp12 Δ2. The amino acid positions of two functional domains, RdRP and the Nidovirus RdRP-associated nucleotidyl transferase (NiRAN), and the deleted regions are indicated. (b) Co-immunoprecipitation of p50 with wild type and IBV nsp12 deletion mutants. HEK293T cells were transfected with wild type and the two deletion constructs, harvested at 24 h post-transfection, lysed with RIPA buffer and subjected to immunoprecipitation with anti-Flag beads. Total cell lysates and precipitates were analysed by Western blot with anti-Flag and anti-Myc antibodies, respectively. Numbers on the left indicate protein sizes in kilodalton.

To further map the region(s) responsible for the interaction between nsp12 and p50, the two conserved sequences in the C-terminal region of IBV nsp12 (Supplementary Figure 3) were deleted, generating two deletion constructs, IBV nsp12 Δ1 and IBV nsp12 Δ2. These two deletion constructs contain deletions of the C-terminal 623–652 and 684–706 amino acid regions, respectively (Figure 6(a)). Transfection of 293  T cells with wild type and the two deletion mutants and analysis by co-immunoprecipitation and Western blot showed much weaker interaction between IBV nsp12Δ2 and p50 (Figure 6(b)). It was also noted that the protein level of IBV nsp12Δ1 was consistently lower than wild type and the other mutant (Figure 6(b)), indicating that this deletion may have a negative impact on the expression and stability of the protein. However, this deletion mutant appears to interact with p50 effectively. Taken together, these results demonstrate that the conserved region from amino acids 684 to 706 may be partially responsible for the interaction between the coronavirus nsp12 and p50.

## Discussion

As intracellular parasites, viruses have evolved various strategies to subvert the host cell cycle to ensure the normal replication of themselves [7]. Depending on the events that occur in each phase of the cell cycle, coronaviruses regulate the cell cycle arrest in different phases according to their own needs for reproduction [22]. We have previously shown that IBV infection of mammalian cells arrested cell cycle in S and G2/M phases by regulating the accumulation of various cyclins and hypophosphorylated RB in a p53-independent manner [10]. Interaction of coronavirus nsp13 with the p125 catalytic subunit of pol δ induced DNA damage response and cell cycle arrest in S phase [12]. In this study, we further demonstrate that coronavirus nsp12 interacts with the p50 regulatory subunit of pol δ, playing a synergistic role with nsp13 in the induction of cell cycle arrest in the S phase.

In addition to the GST-pulldown and co-immunoprecipitation, the interaction of IBV nsp12 with p50 in IBV-infected cells was reinforced by direct visualization of the two proteins co-localized in the infected cells. Determination of the precise subcellular localization of IBV nsp12 in IBV-infected cells was facilitated by the construction and successful rescue of a recombinant IBV harbouring an HA-tagged nsp12. The colocalization of the majority of IBV nsp12 with dsRNA confirms the essential role of this protein in the formation of viral replication/transcription complexes (RTC). Similar to other coronavirus nsps involved in the formation of RTC and induction of double membrane vesicles (DMV), nsp12 may play additional roles in regulating the virus-host interaction. Elucidations of these functions would be greatly facilitated by the construction and rescue of recombinant viruses with a well-defined tag inserted into the protein of interest. As demonstrated in this study, the colocalization of a small portion of the HA-tagged IBV nsp12 with p50 in the nucleus supports its role in regulating cell cycle progression.

Pol δ is mainly involved in the synthesis of the lagging strands during DNA replication. The p50 subunit acts as a scaffold to combine with the p125 catalytic subunit as well as other two subunits [23]. The interaction of coronavirus nsp13 with p125, as reported in our previous study, relocated part of pol δ outside the nucleus and prolonged DNA synthesis time of lagging strands, resulting in the induction of DNA replication stress due to the accumulation of ssDNA and S-phase arrest [12]. In this study, we showed that IBV infection also relocates part of the p50 to the cytoplasm, but this cytoplasmic p50 appears not to be overlapped with nsp12. Instead, the interaction between the two proteins was shown to occur in the nucleus, forming condensed puncta. The underlying mechanism is yet to be illuminated, but the inclusion of a majority of nsp12 in RTC may separate it from p50 in the cytoplasm. The aggregation of p50 by its interaction with the nuclear nsp12 would prevent pol δ from completing the synthesis of the lagging chain, leading to the induction of DNA replication stress and S-phase arrest. The retention of a certain portion of p50 in the cytoplasm would also contribute to the IBV infection-induced cell cycle arrest. Our observation of the minor, although significant, synergistic role in the induction of S phase arrest by co-expression of nsp12 and 13 from three different coronaviruses might be under-representative, as overexpression of either protein may reach a certain threshold, which may limit a further increment. Our previous studies showed that synchronization of Vero and H1299 cells slightly enhanced IBV replication and IBV-induced cell cycle arrest in S and G2/M phases [10,12]. It would be interesting to test in future studies if synchronization of cells would render a similar effect on cell cycle arrest induced by overexpression of the two proteins. We have also noted that the general cell cycle profiles in IBV-infected cells were much different from those in the transfected cells. These differences may be due to the use of serum-free media in the infected cells and serum-containing media in the transfected cells. As addition of serum to the culture medium during or post-viral adsorption would suppress IBV infection of cells and viral replication, serum-free media were used in all infection studies.

In addition to acting as an auxiliary subunit of pol δ, p50 also constitutes an important component of DNA polymerase ζ (pol ζ) together with p66. As auxiliary subunits, p50 and p66 play essential roles in improving the catalytic efficiency and continuity of pol ζ [24,25]. Pol ζ functions as a DNA trans-damage polymerase and also plays a role in normal mammalian cell proliferation; continued failure of pol ζ may lead to DNA breaks and G2 phase arrest [26]. In the study, overexpression of nsp12 induced relatively more cells arrested at the G2/M phase than in cells overexpressing nsp13. It is not sure if this effect is caused by the interaction of nsp12 with pol ζ. Further studies would be required to address this possibility.

Regulation of cell cycle progression appears to be a common strategy exploited by viruses to obtain materials and cellular microenvironments that are conducive to viral replication [27]. Different coronaviruses induce cell cycle arrest in different ways. SARS-CoV accessory proteins 3a and 7b inhibit the phosphorylation of retinoblastoma protein (pRB) by significantly reducing cyclin D3 expression through the cyclin D3/pRb pathway, thereby inducing cellular G0/G1 arrest [28,29]. MHV infection significantly reduced Cdk4, Cdk6, G1 cyclins and pRb phosphorylation in infected cells, and the reduced phosphorylation of pRb suppressed DNA replication and arrested cells in G0/G1 phase [8,30]. TGEV and porcine epidemic diarrhoea coronavirus (PEDV) N proteins were shown to activate p53 and upregulate p21, which in turn inhibits the activation of cdc2/cyclin B1, resulting in cell arrest in G2/M and S phases, respectively [31,32]. Coronaviruses can also manipulate the cell cycle by directly interacting with cell cycle-related proteins. MHV nsp15 interacts directly with pRb and results in S-phase arrest due to downregulation of pRb expression by the endoribonuclease activity of nsp15 [33]. This study adds one more example to the strategies exploited by coronaviruses to regulate the cell cycle for efficient viral replication.

In summary, this study confirms the direct interaction of nsp12 from IBV, SARS-CoV and SARS-CoV-2 with the p50 regulatory subunit of pol δ. Together with the previously identified interaction between coronavirus nsp13 with the p125 catalytic subunit of pol δ, these interactions play synergistic roles in the induction of cell cycle arrest in the S phase. The simultaneous interaction of an essential cellular DNA replication machinery with two core coronavirus replication enzymes highlights the potential of targeting this cellular machinery for antiviral intervention.

## Supplementary Material

Supp_Figures.pptx
