# Supplementary material for: Coronavirus RNA-dependent RNA polymerase interacts with the p50 regulatory subunit of host DNA polymerase delta and plays a synergistic role with RNA helicase in the induction of DNA damage response and cell cycle arrest in the S phase
Source: Emerg Microbes Infect. 2023 Feb 15;12(1):e2176008. doi: 10.1080/22221751.2023.2176008 (PMC9937006; doi:10.1080/22221751.2023.2176008)
Supplement: Supp_Figures.pptx [file TEMI_A_2176008_SM4425.pptx]

## Slide 1
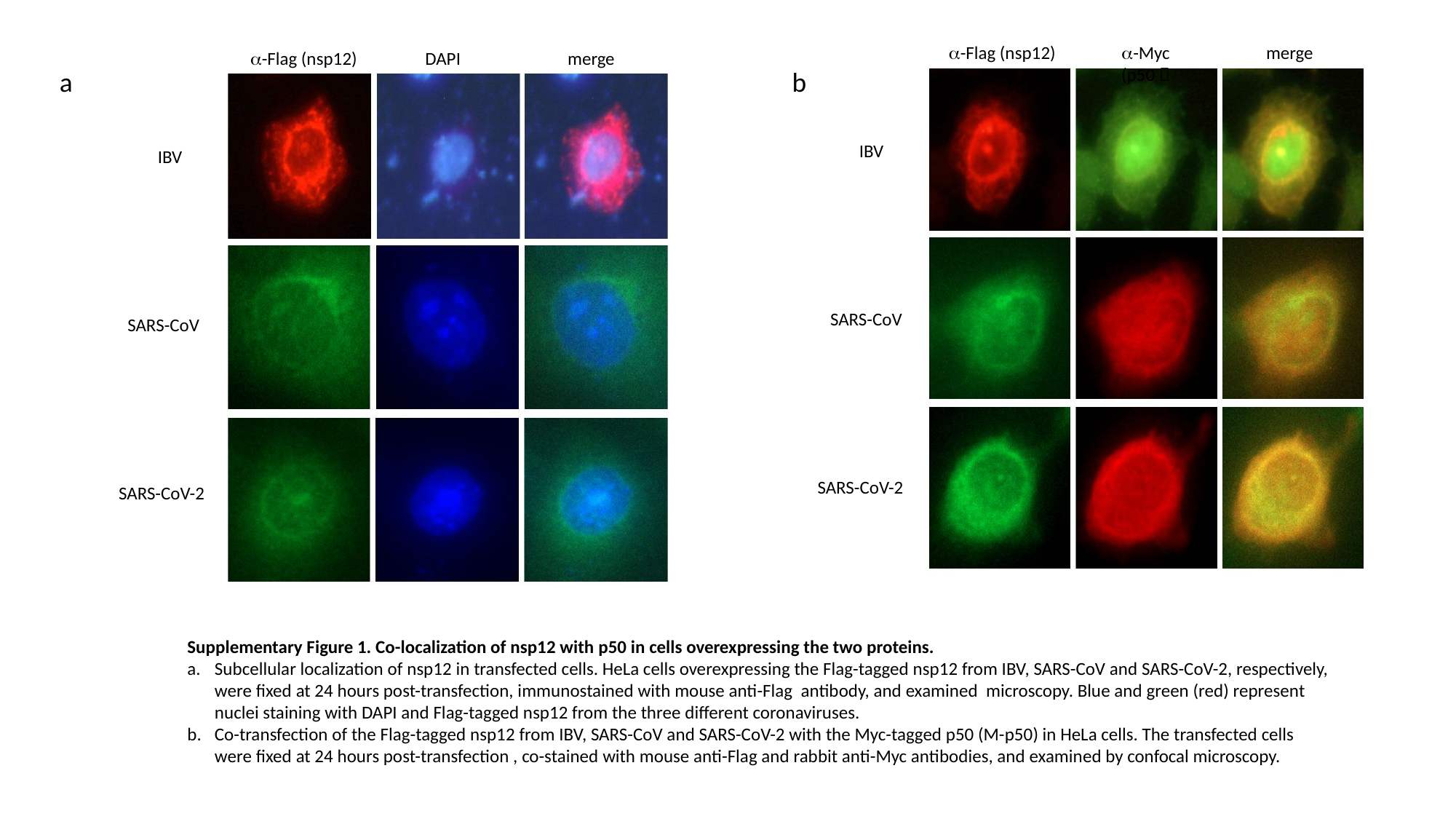

a-Flag (nsp12)
a-Myc (p50）
merge
IBV
SARS-CoV
SARS-CoV-2
a-Flag (nsp12)
DAPI
merge
IBV
SARS-CoV
SARS-CoV-2
a
b
Supplementary Figure 1. Co-localization of nsp12 with p50 in cells overexpressing the two proteins.
Subcellular localization of nsp12 in transfected cells. HeLa cells overexpressing the Flag-tagged nsp12 from IBV, SARS-CoV and SARS-CoV-2, respectively, were fixed at 24 hours post-transfection, immunostained with mouse anti-Flag antibody, and examined microscopy. Blue and green (red) represent nuclei staining with DAPI and Flag-tagged nsp12 from the three different coronaviruses.
Co-transfection of the Flag-tagged nsp12 from IBV, SARS-CoV and SARS-CoV-2 with the Myc-tagged p50 (M-p50) in HeLa cells. The transfected cells were fixed at 24 hours post-transfection , co-stained with mouse anti-Flag and rabbit anti-Myc antibodies, and examined by confocal microscopy.

## Slide 2
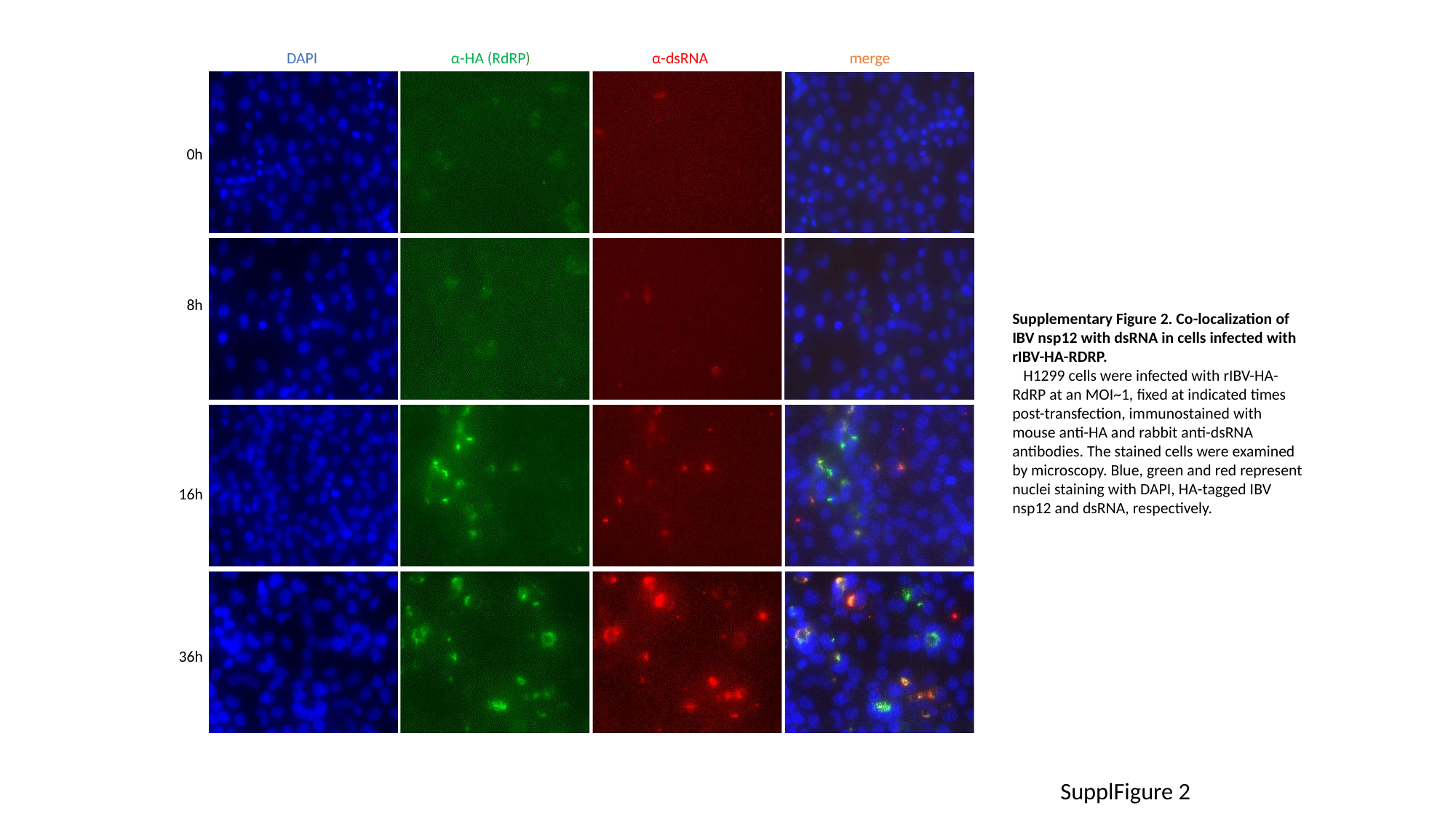

DAPI
α-HA (RdRP)
α-dsRNA
merge
0h
8h
Supplementary Figure 2. Co-localization of IBV nsp12 with dsRNA in cells infected with rIBV-HA-RDRP.
 H1299 cells were infected with rIBV-HA-RdRP at an MOI~1, fixed at indicated times post-transfection, immunostained with mouse anti-HA and rabbit anti-dsRNA antibodies. The stained cells were examined by microscopy. Blue, green and red represent nuclei staining with DAPI, HA-tagged IBV nsp12 and dsRNA, respectively.
16h
36h
SupplFigure 2

## Slide 3
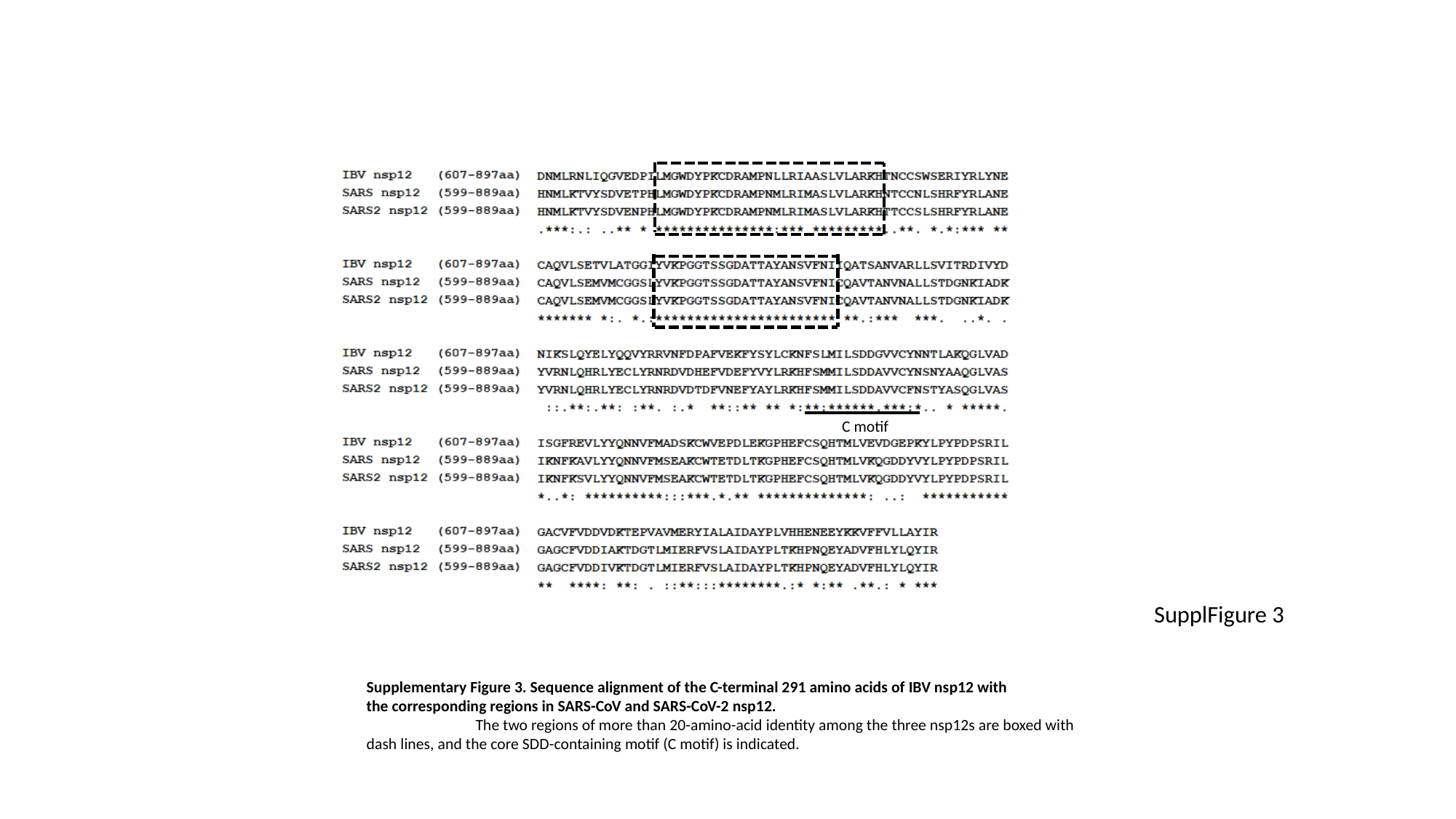

C motif
SupplFigure 3
Supplementary Figure 3. Sequence alignment of the C-terminal 291 amino acids of IBV nsp12 with
the corresponding regions in SARS-CoV and SARS-CoV-2 nsp12.
	The two regions of more than 20-amino-acid identity among the three nsp12s are boxed with
dash lines, and the core SDD-containing motif (C motif) is indicated.
